# Murine mesothelin: characterization, expression, and inhibition of tumor growth in a murine model of pancreatic cancer

**DOI:** 10.1186/s13046-016-0314-2

**Published:** 2016-03-01

**Authors:** Emmanuel Zervos, Steven Agle, Andrew G. Freistaedter, Gwendolyn J. B. Jones, Rachel L. Roper

**Affiliations:** Department of Surgery, Brody School of Medicine, East Carolina University, 600 Moye Blvd, Rm 5E106A mailstop 629, Greenville, NC 27834 USA; Microbiology & Immunology, East Carolina University Brody School of Medicine, Greenville, USA

**Keywords:** Mesothelin, Pancreas, Tumor, Proliferation, Immune

## Abstract

**Background:**

Mesothelin has attracted much interest as a tumor specific antigen; it has been reported to promote tumor development and to be a good target for cancer treatment. Most studies to date have used human mesothelin in immunocompromised mice. Since these models do not allow for study of the natural immune response to mesothelin expressing tumors, we have undertaken the characterization of mouse mesothelin so the effects of this protein can be assessed in immunocompetent mouse strains.

**Methods:**

We analyzed mouse mesothelin expression, tissue distribution, shedding and biochemistry. In addition we constructed stable mesothelin overexpressing lines of the pancreatic cancer line Panc02 by two methods and tested them for growth and tumorigencity in vitro and in vivo.

**Results:**

We show here that mouse mesothelin is similar to human mesothelin in biochemical characteristics, tumor expression and tissue distribution, suggesting the mouse may be a suitable model for study of mesothelin. Stable overexpression of mesothelin in a pancreatic cancer cell line did not increase cell proliferation or anchorage-independent growth in vitro, suggesting that mesothelin is not necessarily a tumor progression factor. Surprisingly overexpression of mesothelin *inhibited* tumor formation in vivo in immunocompetent mice.

**Conclusion:**

The mouse may be a good model for studying mesothelin in the context of an intact immune response. Mesothelin is not necessarily a tumor progression factor, and indeed mesothelin overexpression inhibited tumor growth in immunocompetent mice.

## Background

Human mesothelin is normally expressed only in mesothelial cells of the pleura, peritoneum, and pericardium. However, mesothelin is overexpressed in a high percentage of ovarian, pancreatic, non–small cell lung, and mesothelioma tumors [1–4], and thus is thought to play a role in development of certain cancers. The homeostatic function of mesothelin in mammals is unknown, and the gene can be deleted without apparent effect in mice [5]. Human mesothelin has been proposed to be a malignancy factor as it increased tumor cell proliferation and migration in vitro and tumor size in nude mice [3] and increased cell proliferation in lung adenocarcinomas [6]: mesothelin has also been reported to increase cell invasion [7, 8]. Furthermore, siRNA specific for mesothelin suppressed tumor growth in a rat renal carcinoma model [9]. Mesothelin interacts with CA125/MUC16 ovarian cancer antigen, and this interaction has been hypothesized to play a role in metastasis of ovarian cancers [10].

Since mesothelin expression is largely limited to cancerous cells in the adult, it may also be an effective target tumor antigen for anti-cancer vaccines or therapies [3, 11–14]. Strategies targeting mesothelin have shown some efficacy in phase 1 clinical trials [15]. Both antibodies and T lymphocytes have been shown to have protective anti-mesothelin activity [3, 13, 16, 17]. This fact may be especially useful because irradiation of tumor cells enhances the expression of mesothelin [14], so that the combination of these treatment modalities may be synergistic.

Human mesothelin has been well characterized, and the role of human mesothelin in tumor growth and treatments in immunocompromised mice has been explored [3, 6, 7, 9]. However, these models preclude study of interactions of the tumor with an intact host immune system that may be crucial for its control. Thus, we wanted to characterize mouse mesothelin, to determine its expression, transforming activity, and to develop models in immunocompetent mice to study the role of this important protein. We describe here the expression of mesothelin in murine tissues and cancer cells and show that its overexpression has little effect on cell proliferation or anchorage independence, yet mesothelin overexpression impedes tumor growth in the murine pancreatic adenocarcinoma Panc02 model [18]. Pancreatic cancer is the 5th leading cause of cancer deaths in the United States [19], largely owing to the fact that radiation, surgery, and chemotherapy are ineffective. New treatment strategies are essential, and our data underscore that mesothelin studies should be carried out in vivo in immunocompetent mammals.

## Methods

### Cell lines and antibodies

The C57BL/6 chemically-induced pancreatic adenocarcinoma cell line, Panc02, was a kind gift from Dr. Keping Xie (MD Anderson Cancer Center, Houston, TX). NIH3T3, mouse embryonic fibroblast cell line (CRL-1658), Lewis Lung, and EL4, mouse T lymphoblast thymoma (TIB-39), were kind gifts of Dr. Kathryn Verbanac (East Carolina University, Greenville, NC). OVCAR3 (ATCC HTB-161), human ovarian adenocarcinoma, was the gift of Dr. Anne Kellogg (East Carolina University). TRAMP-C3 murine prostate adenocarcinoma was a gift of Dr. Fred Bertrand (East Carolina University). J774 mouse macrophage (TIB-67), MIA PaCa-2 (CRL-1420), and HPAC (CRL-2119) human pancreatic carcinomas were obtained from ATCC. HEKGP2-293 (Clontech) was derived from HEK cells. Murine mesothelioma AB12 cell line was the kind gift of Dr. Wayne Aldrich, Cleveland Clinic [20]. The JWF2 murine keratinocyte carcinoma cell line was a kind gift from Dr. Rukiyah VanDross, East Carolina University). The rat IgG2a anti-mouse mesothelin monoclonal antibody (clone B35 [10]) was obtained from MBL (Woburn, MA) and the rabbit anti-β-actin antibody was obtained from Cell Signaling Technology. Secondary antibodies for flow cytometry (Cy5 conjugated) and Westerns (HRP-conjugated) were obtained from Jackson Immunoresearch (West Grove, PA).

### RT-PCR to detect mesothelin

Total RNA was isolated from wild type (WT) Panc02 using trizol, and cDNA was synthesized using iScript kit (Biorad, Hercules, CA). Endpoint PCR was performed using the iScript cDNA and puReTaq Ready-To-Go PCR beads (GE Healthcare, Piscataway, NJ) in a Geneamp PCR System 9700. The PCR primers were designed using Integrated DNA Technologies web site. Primers for murine mesothelin were: ACCGACGAGGAACTGAATGCTCTT, and ACGATGGACTCATCCAACACTGCT. Primers to detect GAPDH expression were: AACTTTGGCATTGTGGAAGGGCTC and ACCCTGTTGCTGTAGCCGTATTCA. GAPDH control yielded a product size of 449 and the mesothelin amplicon was 473 bp. The PCR products were run on a 1 % agarose gel and the bands visualized with a Bio Doc-IT camera system (Fig. 2a).

### Flow cytometry

Panc02 cells were washed with DPBS and cells harvested by Trypsin-free Cell Dissociation Solution (Sigma-Aldrich). Cells were incubated in cold flow buffer (DPBS with 0.2 g % BSA + 0.01 % sodium azide) with 1:1000 of MBL B35 rat monoclonal antibody or normal rat IgG for background control. Following 30 minute incubation on ice, donkey anti-rat Cy5 conjugated secondary antibody was added at 1:100. Cells were incubated for an additional 30 minutes on ice, washes repeated and cells were fixed with 1 % electron microscopy grade paraformaldehyde (Electron Microscopy Sciences, Hatfield, PA) diluted in DPBS. Data were acquired on a BD LSR II (BD Biosciences) and data analysis was conducted using BD FACSDiva or FlowJo software. We found that trypsin cleaves mesothelin from the surface, so in experiments with adherent cells, cells were dissociated from the surface using trypsin-free solutions.

### Stable overexpression of mesothelin in Panc02 cells by transfection

Full length mouse cDNA mesothelin sequence was cut from pCMV-SPORT6-meso (OpenBiosystems) by EcoRI and NotI and cloned into EcoRI and NotI sites of pcDNA 3.1 (+) expression vector (Invitrogen). The mesothelin cDNA was sequenced in the pcDNA3.1-meso vector to confirm the correct sequence. Panc02 cells were transfected with pcDNA 3.1 –meso using Lipofectamine 2000 (Invitrogen) following the manufacturer’s instructions. After 48 h transfection pcDNA-meso positive cells were selected with G418 for 10 days (at this time, all the control Panc02 were dead). Clones of Panc02-meso were obtained by limiting dilution. Control Panc02-pcDNA vector cells were obtained by Panc02 cells transfected with pcDNA 3.1 null vector and selected with G418.

### Stable overexpression of mesothelin in Panc02 cells by retroviral (RV) gene transfer

SalI and XbaI were used to cut full length mouse meso cDNA sequence from pCMV-SPORT6-meso, which was cloned into the SalI and AvrII sites of the pLXRN Retroviral Expression Vector (Clontech, Mountain View, CA). Subsequent sequencing of cloned pLXRN-meso confirmed the correct mesothelin cDNA sequence. GP2-293, pantropic virus packaging cell line (Clontech), was cotransfected per manufacturer’s instructions with pLXRN-meso (or pLXRN null vector) and pVSV-G (Clontech), using Lipofectamine 2000 (Invitrogen). Virus-containing supernatants from both cultures of GP2-293 were harvested at 72 h, 0.45 μ filtered, polybrene 1 ug/ml added and used to infect WT Panc02 cells. G418 was added at 48 h post infection to select stably overexpressing or vector control cells, and overexpressing cells were cloned by limiting dilution.

### Rabbit anti mouse mesothelin anti-peptide antibody

**Fig. 1:**
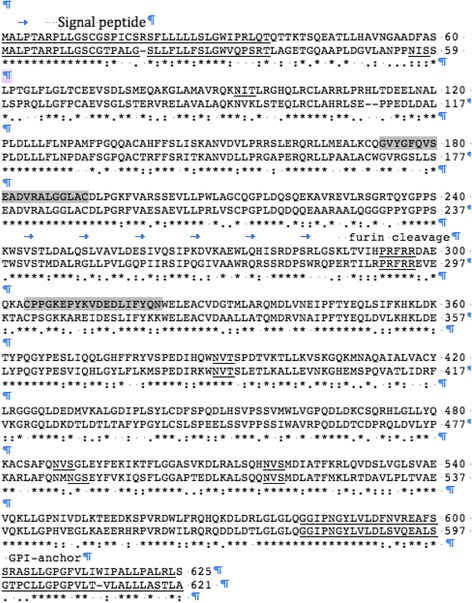
Alignment and high similarity of mouse (*top line*) and human mesothelin protein sequences. Mouse mesothelin (625 amino acids) is predicted to have an amino terminal signal peptide, a furin cleavage site at position 298, and a carboxy-terminal glycosyl-phosphatidylinositol (GPI) linked anchor sequence. Signal peptide, N-linked glycosylation consensus sites, furin cleavage sequence, and GPI-anchor are *underlined*, and peptides used as the immunizing antigen for anti peptide antibody production are *shaded*

Peptides representing murine mesothelin coding sequence, GVYGFQVSEADVRALGGLA**C** and **C**PPGKEPYKVDEDLIFYQN, were synthesized and conjugated to KLH carrier proteins via the terminal cysteine residues (bold, Genemed Synthesis), and used for immunization of two rabbits. Sera were confirmed for reactivity with the immunizing peptides by ELISA. Antibodies were also purified from this sera using peptide CPPGKEPYKVDEDLIFYQN which is in the carboxy portion of the protein, after the furin cleavage site (see Fig. 1).

### Western immunoblot

Panc02, NIH/3 T3, and GP2 HEK-293 cells were lysed in lysing buffer (Tris–HCl 50 mM pH 7.4, sodium deoxycholate 0.25 %, IGEPAL CA-630 1 %, NaCl 150 mM, EDTA 1 mM, EGTA 1 mM) containing 1× HALT Protease Inhibitor cocktail (Pierce Chemical, Rockford, IL) and centrifugation at 14,000 × g for 15 minutes. Supernatant was collected and a BCA Assay (Pierce Chemical) performed to quantify total protein. Tissues from C57Bl/6 mice were harvested and homogenized in lysis buffer with 1× HALT at 100 mg tissue/ml buffer using a Polytron PT 1200E. Homogenized samples were centrifuged, supernatants harvested and protein levels determined by BCA assay as above. Total proteins (40 μg/lane in sample buffer containing 0.1 M dithiothreitol) were subjected to electrophoresis on 8 % SDS-acrylamide gels. Proteins were transferred to PVDF membranes in Towbin’s transfer buffer for 75 min at 175 mA (XCell II Blot Module, Invitrogen) and blocked in 5 % powdered milk in TBST. Blots were incubated with a 1:8000 dilution of rabbit anti-mouse mesothelin polyclonal antisera in 5 % milk/TBST overnight at 4 °C. Blots were incubated with donkey anti-rabbit IgG peroxidase-conjugated antibody (1:8,000) and proteins visualized by luminol-enhanced chemiluminescence using Lumiglo ECL reagent (NEB) and exposure of membranes to x-ray film (Hyperfilm ECL, GE Healthcare Amersham). For detection of mesothelin overexpression with pcDNA or RV in the 6 clones, 4, 1.8 × 10^6^ Panc02 cells were lysed with 200 ul 1× disruption buffer (4 % SDS, 4 % ß-mercaptoethanol, 0.05 M Tris base, 10 % Glycerol 0.1 % Bromphenol blue). The cell lysate was passed through 22 Gauge needle and 26 ul lysate was heated at 100 °C for 10 min and loaded on an 8 % SDS gel and processed for western blot as described above. For measurement of actin, the blot was stripped with BlotFresh Western Blot Stripping Reagent (SignaGen Lab) and incubated with 1:1500 dilution of rabbit anti actin antibody (Cell signaling,) followed by donkey anti-rabbit IgG peroxidase-conjugated antibody (1:8000) and development by chemiluminescence. For Western analysis on supernatants, cells were cultured in RPMI 1640 medium supplemented with 1 % of FBS, 1 mM of sodium pyruvate, 100 uM of NEAA 0.05 mM of ß-mercaptoethanol and 300 ug/ml of Geneticin (GIBCO). One day later, the supernatants were harvested and 16:1 concentrated using Millipore 10 kDa Centrifugal Filter Units. BCA assay was used to measure the protein concentration of the concentrated supernatant. 50 ug of the supernatant protein was mixed with sample loading buffer with 0.1 M dithiothreitol, heated at 100 °C for 10 minutes, loaded on a 10 % SDS gel and processed for Western blot as above.

### Tumor model

Female C57BL/6 mice (Charles River, 10 and 14 weeks of age) were used for experiments. All mice were housed in the East Carolina University Department of Comparative Medicine AAALAC accredited animal facility and kept in conventional conditions with full access to food and water throughout the study. All procedures were approved by the Institutional Animal Care and Use Committee and in accordance with recommendations for proper care and use of laboratory animals. Groups of mice (*n* = 4–8 per group) were injected s.c. in the right flank with Panc02 cells (6.4x10^5^), one injection per mouse, under isoflurane (3 %) inhalation anesthesia. Tumors were allowed to grow until palpable, and tumor size was measured three times per week by a digital caliper, and volume was calculated as [(smallest diameter^2^) x largest diameter]/2. Mice were humanely euthanized to minimize pain or distress if they reach endpoints of tumor size, ulceration, or impairment.

### Panc02 in vitro growth MTS assay

Panc02 cells were seeded in 96-well plates (15,000 cells/100ul/well), at serum concentrations from 0-10 %, and after 24 h, 10 μl of MTS/PMS (2.0 mg/ml MTS, Promega and 0.1 mg/ml PMS, Sigma) was added. The absorbance was read at 492 nm. Media only was used to define the background control level. In another experiment, 100 cells/well were seeded into wells of a 6 well plate and allowed to grow for 10 days. MTS/PMS was added and readings were performed as above.

### Soft agar assay

A base layer of 1 m1 of 0.5 % agar in RPMI was poured into wells in a 6 well plate and cooled to solidify. 2 ml of warmed (40 °C) 0.2 % agarose/RPMI cell solution (25,000 cells per ml) were layered on top of each well. Cells were incubated at 37 °C 5 % CO2 for 3 weeks. 500 ul of RPMI per well were added once per week. Wells were photographed macro-and microscopically for colony counting and measurement.

### Statistical analysis

Statistical analysis consisted of ANOVA using the 5 groups (wild type, vector, and 3 clones) for both the pcDNA and the RV vectored mesothelin overexpressing clones with post hoc testing with a Tukey adjustment. Students t test was used to assess significance between vector and mesothelin overexpressing lines.

### Tumor section staining

Frozen tissue was embedded in Frozen Section Medium (Azer Scientific), cut on a cryostat (5 um), and mounted onto Superfrost Plus glass microscope slides. Tissue was fixed on slides with acetone (−20 °C) and endogenous peroxidase activity was blocked with 0.6 % hydrogen peroxide. Slides were placed on Shandon coverplates™ (Thermo Fisher) using 0.2 % Triton-× 100/PBS and placed in a Sequenza™ slide rack (Thermo Fisher). Slides were incubated with Biotin conjugated rat anti-mouse CD8 (eBioscience, 1:50 in 1%BSA/PBS), or diluent as negative control, and then with Vectastain Elite ABC (Vector Laboratories, Burlingame, CA), developed with Vector DAB Peroxidase substrate (Vector Laboratories) and mounted with Permount (Thermo Fisher).

## Results

### Mouse mesothelin

Figure 1 shows the alignment and high similarity of mouse (top line) and human mesothelin protein sequences. Mesothelin is highly conserved, and BLAST-p shows that mouse mesothelin shares 92 % identical or conservative substitutions to rat mesothelin, 71 % to human mesothelin, 70 % to rhesus monkeys, and 74 % to dog. Mouse mesothelin is predicted to have an amino terminal signal peptide, a furin cleavage site at position 298, and a carboxy-terminal glycosyl-phosphatidylinositol (GPI) linked anchor sequence also conserved in the rat and human orthologs [21]. Similar to human and rat sequences, the murine mesothelin sequence is predicted to be 625 amino acids, which would result in a mature protein of approximately ~65 kDa (if the putative signal sequence is cleaved). Furin cleavage of mouse mesothelin would be predicted to result in a released amino terminal fragment of ~29 kDa and a membrane-bound carboxy terminal fragment of ~37 kDa. However, since each of these 2 major fragments has N-linked glycosylation sites (1 in the soluble amino terminal fragment and 3 in the membrane bound form) in human and mouse, the glycosylation patterns determine the actual molecular weight of the proteins expressed in cells. Interestingly, in the human, the carboxy terminal portion of mesothelin (from the furin cleavage site to the gpi linkage) is also reported to be released from the cell surface [22, 23].

### Mesothelin surface expression

**Fig. 2:**
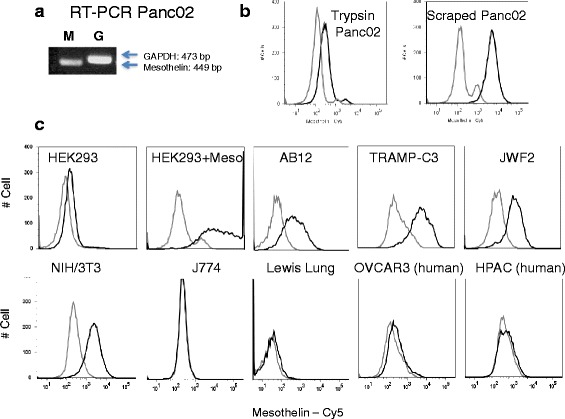
Expression of murine mesothelin. **a** RT-PCR detection of mesothelin (M, 449 base pairs) and control GAPDH (G, 473 bp) RNA in Panc02 cells. **b** Mesothelin protein surface expression – cells were stained with rat anti-mouse mesothelin monoclonal antibody B35 (*black line*) or normal rat IgG (*gray line*) and Cy5-conjugated donkey anti-rat IgG and analyzed by flow cytometry. Top right panels show that Panc02 surface detection is reduced by trypsin treatment compared to scraping of the adherent cells into solution. Adherent Panc02 cells were harvested by treatment with Trypsin/EDTA cocktail or by scraping. **c** Because trypsin reduced mesothelin detection, all subsequent experiments on adherent cells were performed using chelating agents without trypsin. Mesothelin expression was detectable on mouse-mesothelin-transfected HEK, and several mouse cancer cell lines: AB12 mesothelioma, TRAMP-C3 prostate adenocarcinoma, the JWF2 keratinocyte squamous cell carcinoma, and NIH/3 T3 cells

Since there was a dearth of characterized murine mesothelin reagents available, we first assessed murine mesothelin expression using sequence information and RT-PCR. Mesothelin is reported to be over-expressed in pancreatic cancer cells, so we assessed mesothelin expression in the Panc02 murine pancreatic adenocarcinoma line. We were able to detect mesothelin RNA expression in these cells by RT-PCR (Fig. 2a).

Protein sequence analysis suggested that murine mesothelin would be expressed on the surface of cells similar to human mesothelin. In order to detect surface protein expression of mesothelin, we assessed the B35 monoclonal antibody made to LO cells (murine embryonic endothelial-like cells) that was suggested from functional studies to bind murine mesothelin [10]. This antibody recognized a protein on the surface of Panc02 cells, and the protein was removed from the surface by trypsin (Fig. 2b). Therefore, subsequent flow cytometry experiments were performed with cells that were scraped or dislodged by metal ion chelation to avoid the use of trypsin. To determine the specificity of the B35 antibody for mouse mesothelin, we performed a transfection experiment. Transfection of HEK293 cells with murine mesothelin resulted in >100 fold increase mean fluorescence intensity compared to vector transfected cells. These data constitute the first direct evidence that the B35 antibody detects mesothelin (Fig. 2c).

We next wanted to characterize the expression of mesothelin in various mouse cell types. As shown in Fig. 2c, mesothelin expression was detectable on several mouse cancer cell lines: AB12 mesothelioma, TRAMP-C3 prostate adenocarcinoma, and the JWF2 keratinocyte squamous cell carcinoma. It was also detected on MEF and NIH/3 T3 embryonic fibroblast lines, consistent with previous detection during embryogenesis [5]. However we found for the mouse, as in humans, mesothelin protein expression was largely confined to certain cancers. A number of murine cell lines did not express mesothelin, including: P815 mastocytoma, YAC-1 and EL4 T lymphomas, B16-F10 melanoma (not shown), Lewis lung carcinoma, the J774 macrophage/monocyte cell line (Fig. 2c). In addition the B35 antibody did not detect mesothelin on any human cells tested; HEK-293, OVCAR3, and HPAC (Fig. 2c) and MIA PaCa2 (not shown). Since OVCAR3 and HPAC cells are reported to be positive for human mesothelin, these data suggest that the B35 antibody is specific for murine mesothelin. Thus, our data show that the distribution of murine mesothelin is limited to certain cancers similar to human mesothelin expression and suggest the use of certain mouse tumor models for the study of mesothelin.

### Mesothelin expression in mouse tissues

**Fig. 3:**
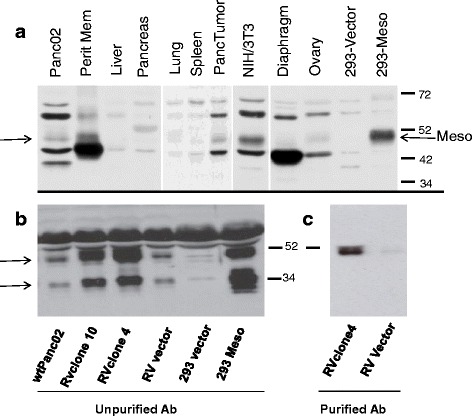
Mesothelin expression in normal mouse tissues and cell lines. **a** Cell lines and naive C57Bl/6 mouse tissues (including peritoneal membrane, pancreas and diaphragm) were harvested, prepared and assayed as described in Methods. Samples were subjected to denaturing electrophoresis on 8 % SDS-acrylamide gels. Immunoblots were probed with polyclonal affinity-purified rabbit anti-mouse mesothelin antibody and HRP-conjugated donkey anti-rabbit antibody. *Arrows* point to mesothelin protein bands **b** Mesothelin can be detected by unpurified rabbit anti mesothelin antisera in supernatants of Wt Panc02 cells, with increased levels in Panc02 cells containing retrovirus constructs (RV) over-expressing mesothelin (RV clone 4, RV clone10), compared to Panc02 containing empty vector (RV vector). Mesothelin can also be seen in HEK 293 cells transfected with a mesothelin expressing plasmid (293 Meso). **c** Mesothelin can be detected in supernatants (RVclone 4) using sera purified on peptide PPGKEPYKVDEDLIFYQN in the carboxy terminal half of the mesothelin protein. The purified antibody detects the 49 kDa form of mesothelin, but not the 34 kDa form

It was reported that murine mesothelin gene expression was detected in certain embryonic stages as well as in several adult tissues, but protein expression was not tested previously [5]. While the B35 anti-mesothelin antibody did not work in a denaturing western blot, we were able to detect mouse mesothelin by preparing an affinity purified polyclonal rabbit antibody. Mesothelin was detected as a ~49 kDa protein in some mouse tissues and in HEK 293 cells transfected with a mesothelin expressing plasmid (Fig. 3a). The apparent molecular weight of the mesothelin protein is consistent with the predicted molecular weight of the furin-cleaved mature glycosylated protein (37 kDa plus glycosylation). A full-length precursor mesothelin protein at the predicted 66 kDa (plus glycosylation) was not clearly visible on gels suggesting that the furin cleavage is rapid. Consistent with the flow cytometry data (Fig. 2), we detected mesothelin in Panc02 and NIH 3 T3 cells (Fig. 3a). Mesothelin protein was also detected in Panc02 tumor excised from a mouse, and in normal mouse peritoneal membrane and ovary, as previously described in human tissue [24]. Relative protein expression levels in the different tissues may be inferred from comparison to background staining bands on the blot. HEK 293 cells, and normal mouse diaphragm, spleen, lung, pancreas and liver did not express substantial levels of mesothelin, underscoring the restricted expression of this protein in mice. These data indicate that murine mesothelin expression is similar to human in that it is not broadly expressed on many tissue types, suggesting that mesothelin may be an appropriate tumor-specific target for treatment.

### Mesothelin release

Mesothelin has been reported to be released from cells expressing it [22, 23], so we measured mesothelin in supernatants of cultured Panc02 cells. Figure 3b shows that wild type (wt) Panc02 cells and HEK 293 cells transfected with a pcDNA plasmid expressing mesothelin have a protein supernatant band at ~49 kDa (similar to mesothelin found in lysates) as well as a ~34 kDa form that reacts with the rabbit anti mesothelin antisera we prepared. We constructed Panc02 cells that over-express mesothelin using a retroviral (RV) construct, and both the 34-and 49 kDa proteins were greatly increased in supernatants from these cells (RV clone 4 and 10, Fig. 3b).

As the identity of the secreted mesothelin fragments has been controversial, we also purified rabbit antisera on peptide **C**PPGKEPYKVDEDLIFYQN to purify antibodies reactive with the carboxy portion of the furin-cleaved protein (from the furin cleavage site to the GPI anchor region). This purified antibody recognized the 49 kDa supernatant protein but not the 34 kDa protein (even after extended exposure, Fig. 3c). These data suggest that 2 forms of the mesothelin protein are found in supernatants of murine mesothelin expressing cells, a 49 kDa form that contains sequence from the furin cleavage site to the GPI anchor region, and a 34 kDa form that does not contain the PPGKEPYKVDEDLIFYQN (Fig. 1), presumably the amino terminal secreted form.

### Mesothelin overexpression

**Fig. 4:**
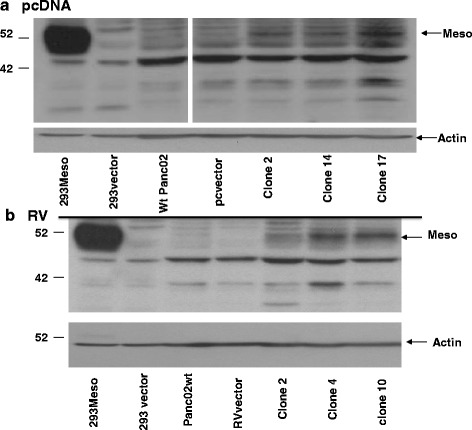
Overexpression of mesothelin. **a** pcDNA- Clones of Panc02 cells (clones 2, 14 and 17) stably transfected with pcDNA mesothelin under the CMV promoter show higher levels of mesothelin expression than wild type Panc02 cells and empty pcDNA vector Panc02 cells. HEK293 cells transiently transfected with empty vector or mesothelin expressing pcDNA vector (293Meso) are shown as control. **b** Overexpression of mesothelin is seen in stably transduced retrovirus (RV) clones of Panc02 (clones, 2, 4, and 10), compared to retrovirus vector. HEK 293 cells transiently transfected with mesothelin over-expressing plasmid or empty vector are shown as controls

In order to study the biology of mesothelin, we constructed stable mesothelin over-expressing Panc02 cell clones by transfection with pcDNA under the CMV promoter followed by drug selection. Figure 4a shows that we were able to over-express mesothelin in 3 clones (2, 14, and 17) compared to wild type (wt) Panc02 and control pcDNA empty vector transfected cells. Panc02 cells show a family of bands around 49 kDa, which are likely glycoyslation variants of mesothelin. The mature protein was visible at similar apparent molecular weight in both Panc02 cells and transfected HEK 293 cells. In order to study mesothelin over-expression in a vector-and promoter-independent manner, we also stably overexpressed mesothelin in Panc02 by retroviral (RV) transduction under the LTR promoter. Figure 4b shows that three clones overexpressed mesothelin compared to wt Panc02 and control empty vector treated cells. There is a similar grouping of bands visible around 49 kDa as well as some smaller bands that react with this antibody.

### Mesothelin overexpression on the surface of cells

**Fig. 5:**
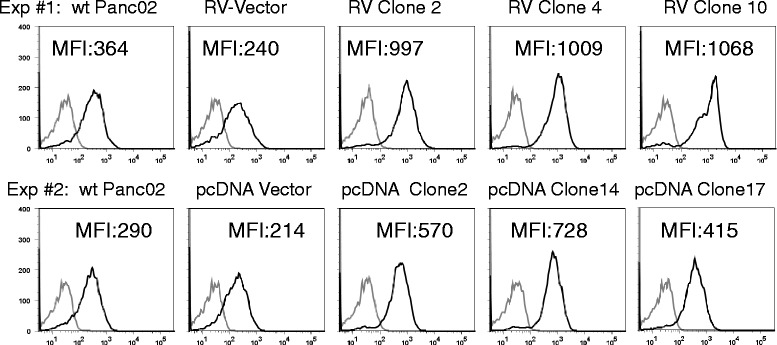
Mesothelin expression on the cell surface. Panc02 wild type (wt), retrovirus (RV), clones 2, 4, 10; or pcDNA clones 2, 14, and 17, or control null vector cells were incubated with monoclonal antibody B35 (*black line*) or normal rat IgG (*gray line*) and Cy5-conjugated donkey anti-rat IgG and analyzed by flow cytometry. Mean fluorescence intensities (MFI) show increased mesothelin in overexpressing clones. RV clones expressed significantly more mesothelin than vector and wild type *p* < 0.001. pcDNA clones 2 and 14 expressed significantly more mesothelin than vector and wild type *p* < 0.05

In order to determine whether over-expressed mesothelin is displayed on the surface of cells, we performed flow cytometry. Figure 5 shows that all 6 clones that over-expressed mesothelin by western blot also over-expressed mesothelin on the cell surface compared to wt Panc02 and empty vector control cells. Mean fluorescence intensities increased 2–3 fold in the mesothelin over-expressing cells. Thus, these data confirm the results in Fig. 4 and indicate that mesothelin was also expresed on the cell surface where it might more easily stimulate or interact with an immune response.

### In vivo tumor kinetics

**Fig. 6:**
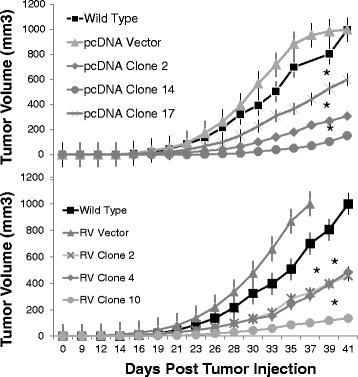
Tumors in mice. C57BL/6 mice (*n* = 4–8 per group) were injected in the flank with 640,000 wild type Panc02 cells, mesothelin overexpressing stable Panc02 cells constructed using retrovirus (RV, clones 2, 4, 10) or pcDNA transfection (clones 2, 14, and 17) or control null-vector containing Panc02 cells. Tumor volumes were measured 3 times per week. Standard error bars are shown and Student's t-test showed statistical significance *p < 0.01 for all 6 overexpressing clones after day 24. ANOVA showed significant differences (*p* <0.05) between 1) the wild type and vector groups and 2) the 3 RV mesothelin overexpressing clones on days 30–39 (except for day 35): and for the pcDNA clones between 1) the wild type and vector groups, and 2) the 3 pcDNA mesothelin-overexpressing clones on days 30–41.

Since human mesothelin has been reported to be an aggression/malignancy factor, we wanted to determine the effect of murine mesothelin overexpression in Panc02 cells. Three stably transfected mesothelin over-expressing clones (CMV promoter) were compared to vector and wild type Panc02 injected s.c. for tumor growth kinetics. Results showed that all mesothelin-overexpressing clones grew tumors significantly more slowly in immunocompetent syngeneic mice than either wild type Panc02 or pcDNA empty vector control (Fig. 6, top). To determine whether mesothelin overexpression reduced tumor growth or if this might be due to the particular promoter or plasmid construct, we also assessed mesothelin ing Panc02 clones constructed using the retroviral (RV) transduction system with mesothelin under the LTR promoter. Results were similar: all three RV mesothelin over-expressing clones tested developed tumors more slowly in mice than either wild type or vector control (Fig. 6). Student’s t-test showed statistical significance *p* < 0.01 for all 6 overexpressing clones (compared to corresponding vector) after day 24. ANOVA showed significant differences (*p* <0.05) between 1) the wild type and vector groups, and 2) the 3 RV mesothelin overexpressing clones on Days 30–39 (except for day 35): and for the pcDNA clones between 1) the wild type and vector groups, and 2) the 3 pcDNA mesothelin-overexpressing clones on days 30–41. It should be noted that both the vector groups and the overexpressing clones express the neomycin resistance gene and were selected on G418, so differences are not attributable to neomycin expression. These results suggested that increasing mesothelin expression in Panc02 cells did not accelerate tumor growth as expected [3], and in fact inhibited it

### Panc02 growth kinetics in vitro

**Fig. 7:**
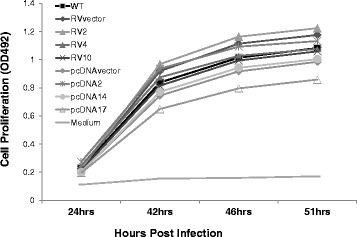
Mesothelin effect on in vitro growth. Wild type (WT) Panc02, mesothelin over-expressing stable Panc02 cells constructed using retrovirus (RV, clones 2, 4, 10) or pcDNA transfection (clones 2, 14, and 17) or control null vector containing Panc02 cells were seeded in triplicate in 96-well plates (15,000 cells/100ul/well) with 10 % serum. After 24 h, 10 μl of MTS/PMS was added to measure metabolism/proliferation, and the absorbance was read at 492 nm

To determine whether the slower growth rate of the mesothelin over-expressing clones seen in vivo was due to the inherent growth rate of the clones or another mechanism of control of tumor growth in vivo, we tested the proliferation of wtPanc02, empty vector transfected, or mesothelin over-expressing Panc02 clones in vitro. The empty vector control cells contain the neomycin resistance gene and were selected on G418 similar to the selection of the clones. As shown in Fig. 7, the growth rate (slope) of all Panc02 cells was similar, with the wild type Panc02 cells in the middle of the group of cells with some mesothelin over-expressing clones showing higher and some showing lower proliferation (the results depend on the exact number of cells counted and plated at the beginning of the experiment). There was no clear trend, suggesting that the level of mesothelin expression did not itself affect the rate of proliferation. This suggests the mesothelin overexpression did not have toxic effects in the cells. In five experiments with various cell and serum concentrations, out to 10 days of growth, no consistent significant mesothelin-dependent growth effect was detected. These results indicated that the Panc02 cells and the mesothelin overexpressing clones grew at a similar rates in vitro and suggest that the growth rate of the mesothelin overexpressing clones (Fig. 6) was slowed significantly by some in vivo effector in the mouse tumor model where there is an intact immune system.

### Anchorage independent growth

**Fig. 8:**
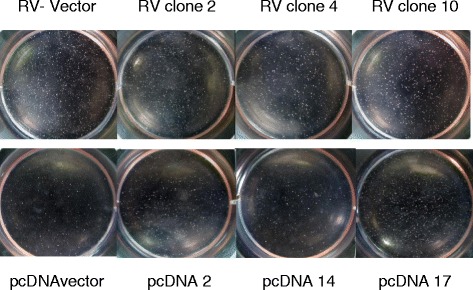
Soft agar assay. A base layer of 1 ml of 0.5 % agar in RPMI was poured into wells in a 6 well plate and cooled to solidify. 2 ml of warm (40 °C) 0.2 % agarose/RPMI cell solution (25,000 cells per ml) were layered on top of each well. Cells (vector control or corresponding mesothelin-overexpressing clones) were incubated at 37 °C 5 % CO2 for 3 weeks. 500 ul of RPMI per well were added once per week. Wells were photographed macro-and microscopically for colony counting and measurement

It has also been previously reported that mesothelin promoted anchorage independent colony formation in vitro [3], considered an in vitro measure of transformation. We analyzed Panc02 wild type, null control vector, and mesothelin over-expressing clones for colony formation in soft agar. We found that the six mesothelin over-expressing clones (3 retrovirus and 3 pcDNA constructs) did not reproducibly form higher numbers of colonies or larger colonies than vector controls (Fig. 8). While there was variability in individual experiments with some clones higher or lower than vector control, the mesothelin overexpressing clones usually formed fewer colonies compared to vector controls when counted microscopically, but there were no statistically significant differences. Together these data indicate that mesothelin overexpression does not promote cell proliferation or anchorage independent growth of Panc02 cells.

### Tumor histology and metastases

**Fig. 9:**
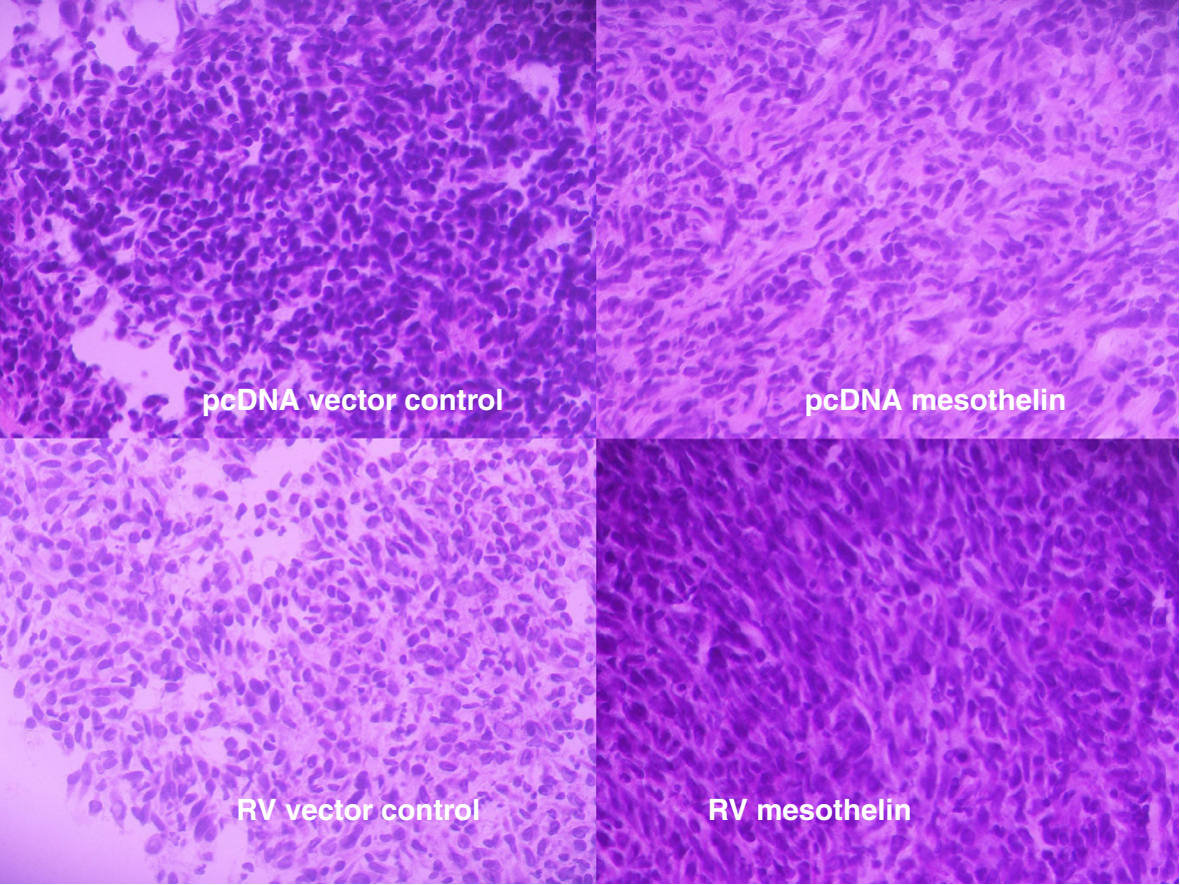
Tumor cell morphology. Control vector (pcDNA plasmid and retroviral vector [RV]) and mesothelin overexpressing tumors harvested from mice (Fig. 6) were frozen, fixed, and stained with hematoxylin and eosin. Vector control tumors (*left*) show epithelial cobblestone morphology while mesothelin over-expressing tumor cells (*right*) show more spindle shaped morphology

In order to view the effects of mesothelin overexpression in vivo, we performed hematoxylin and eosin staining of tissue sections from both pcDNA and retroviral vector mesothelin-overexpressing tumors and control vector tumors (Fig. 9). Epithelial type tumor cells were dominant in all tumors, with viable tumor cells and vasculature throughout and little visible necrosis. Little evidence of leukocytic infiltration was seen in mesothelin-overexpressing or vector control tumors. The mesothelin over-expressing tumors showed an apparent increase in epithelial to mesenchymal transition. The vector control tumors displayed the typical epithelial cobblestone appearance, while over-expression of mesothelin caused more spindle-shaped mesenchymal type cell morphology. The tumor cells overexpressing mesothelin also appeared to have increased production of extracellular matrix (Fig. 9).

**Fig. 10:**
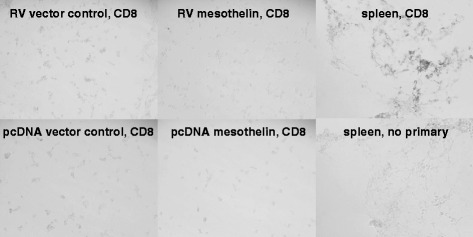
Control vector (pcDNA plasmid and retroviral vector [RV]) and mesothelin overexpressing tumors harvested from mice (Fig. 6) were frozen, fixed, and stained with biotin conjugated rat anti-mouse CD8, Vectastain Elite ABC and developed with Vector DAB Peroxidase substrate. Spleen cells (positive control) show strong staining of CD8+ cells compared to negative control (no primary antibody) or tumor tissue sections

To determine whether there was increased specific immune response to mesothelin, we performed immunohistochemical staining for CD8, a marker for cytotoxic T lymphocytes. As in the hematoxylin staining, little lymphocytic infiltration was seen in the tumors, and there was little CD8 antibody signal compared to control sections with no primary antibody. Spleen cells were stained as positive controls and showed strong CD8 detection. There was no observable difference in CD8 signal between control vector tumors and mesothelin overexpressing tumors (Fig. 10).

Since the epithelial to mesenchymal transition is associated with increased metastasis, we macroscopically analyzed lung metastases in mice with control vector tumors and mesothelin over-expressing tumors. In the vector control tumor-implanted mice, 5 of 12 mice had lung metastases (42 %), while in mesothelin over-expressing tumor-implanted mice, only 2 mice of 26 had metastases (8 %). Additionally, the numbers of metastatic foci per mouse were reduced in mice with mesothelin over-expressing tumors (4 metastases in 2 of 26 mice) compared to control tumors (25 metastases in 5 of 12 mice).

## Discussion

The mouse mesothelin sequence is very similar to human and rat, conserving all the main predicted structural features of the protein, suggesting that its functions and biochemistry are similar [21]. The murine mesothelin sequence is predicted to be 625 amino acids, with a precursor protein of ~65 kDa (plus glycosylation), and furin cleaved fragments of ~29 kDa (predicted to be secreted) and ~37 kDa membrane-bound, which may be shed from the surface. Our results show that murine mesothelin can be detected as a ~49 kDa protein on SDS-PAGE immunoblot, with a 34 kDa band also apparent in some samples. This is consistent with the predicted molecular weights of the furin-cleaved 29 and 37 kDa fragments, since each of these fragments is predicted to be glycosylated. We have shown that mouse mesothelin also has similar expression to human mesothelin, that it is restricted to certain organs and cancer and embryonic cells, and that murine mesothelin is expressed on the cell surface as well as released into supernatants, similar to human cells. The similarities in biochemistry and expression suggest that mouse mesothelin will make an appropriate model for studying mesothelin biology and immunology.

Both carboxy and amino terminal fragments of mesothelin have been reported to be released from cells and are detectable in supernatants and sera of tumor bearing mice or humans [21–23, 25], it seems likely that the 49 kDa form represents the carboxy terminal fragment and the 34 kDa fragment is the amino terminal portion of the mesothelin, however it is possible that the 34 kDa form is a degradation product missing the amino acid sequence used to elicit the antisera (Fig. 1). The smaller amino terminal fragment is predicted to be secreted because of its signal sequence. The carboxy terminal fragment is predicted to be membrane bound by gpi anchor, however gpi proteins can be shed from the cell surface by cleavage by several proteases. The biological effects of the shed mesothelin are unknown, but these fragments may be useful for diagnostic purposes, or they might interfere with mesothelin-specific immune responses.

Human and mouse mesothelin expression have been implicated in the development of cancer [3, 26, 27]. To understand the role that mesothelin plays in cell transformation, we constructed stable mesothelin over-expressing Panc02 clones using both retroviral and pcDNA stable transfection methods. We determined that mesothelin overexpression (in 6 clones) did not increase cell proliferation in vitro or transformation as measured by the soft agar colony formation assay (Figs. 7, 8). In addition, mesothelin overexpression reliably caused a decrease in a heterotopic tumor growth in an immunocompetent syngeneic mouse model, when compared to wild type or stable vector transfected/transduced Panc02 lines. Other cell lines should be tested for effects of mesothelin expression levels since perhaps Panc02 cells already express a high/saturating level of mesothelin, but our data show that the NIH 3 T3 cells express higher mesothelin levels (Fig. 3). Our data are in contrast to data reported on human mesothelin in the MIA PaCa-2, human tumor cell line expressing tumors in nude mice [3], and on mouse mesothelin in nude mice [26] where mesothelin overexpression correlated with increased tumor growth. In our mouse model, the immune system is intact, and since both B and T lymphocyte responses to mesothelin have been shown to have some protective efficacy [3, 13, 16, 17, 28, 29], these data suggest there may be some immune interaction in vivo that contributes to the control of tumor growth.

Analysis of tumor sections however did not show an increase in leukocytic infiltration or CD8+ cells in mesothelin over-expressing tumors (Fig. 10), but did show a clear increase in epithelial to mesenchymal transition (EMT, Fig. 9). EMT is a complex process controlled by the interplay between tumor cells and the microenvironment resulting in a change in tumor phenotype, gene expression, cytokine production, and resistance to apoptosis and immune mediated effects [30–32]. Typically EMT is thought to be associated with an increase in metastasis, but we did not detect any increase in metastasis in these mice. EMT has also been reported to cause a decrease in tumor cell proliferation [33], perhaps affecting the rate of tumor formation in our mesothelin overexpressing tumors. A myriad of cytokines, chemokines, stromal, and innate and adaptive immune cells interact to create the tumor microenvironment in vivo: how mesothelin affects these complex interactions and tumor growth requires further study. Our research underscores the importance of evaluating tumor formation and growth in immunocompetent mouse models where all components of the natural microenvironment can be assessed.

## Conclusions

Mouse mesothelin is similar to human mesothelin in biochemical characteristics, tumor expression and tissue distribution. Stable overexpression of mesothelin in a pancreatic cancer cell line did not increase cell proliferation or anchorage-independent growth in vitro, suggesting that mesothelin is not necessarily a tumor progression factor as previously reported. Overexpression of mesothelin *inhibited* tumor formation in vivo, but not growth in vitro. These data support further research into the effects of mesothelin expression on tumor development.

## Abbreviations

BCA: bicinchoninic acid
CMV promoter: cytomegalovirus promoter
DPBS: dulbecco’s phosphate-buffered saline
EDTA: Ethylenediaminetetraacetic acid
EGTA: ethylene glycol tetraacetic acid
GAPDH: glyceraldehyde 3-phosphate dehydrogenase
gpi: glycosyl-phosphatidylinositol
HRP: horseradish peroxidase
LTR: long terminal repeats
MFI: mean fluorescence intensity
MTS/PMS: tetrazolium compound (3-(4,5-dimethylthiazol-2-yl)-5-(3-carboxymethoxyphenyl)-2-(4-sulfophenyl)-2H-tetrazolium, inner salt/phenazine methosulfate
pcDNA: plasmid vector Invitrogen
PVDF: Polyvinylidene fluoride
RT-PCR: reverse transcriptase polymerase chain reaction
RV: retroviral vector
s.c.: subcutaneous
siRNA: small interfering RNA
TBST: tris buffered saline with tween® 20
Wt: wild type panc02
